# A Novel STAG2 Frameshift Variant in Mullegama–Klein–Martinez Syndrome with Complex Conotruncal Heart Defect

**DOI:** 10.3390/genes16111364

**Published:** 2025-11-10

**Authors:** Hua Wang

**Affiliations:** Division of Genetics, Department of Pediatrics, Loma Linda University School of Medicine, Loma Linda, CA 92350, USA; huawang@llu.edu

**Keywords:** *STAG2*, Mullegama–Klein–Martinez syndrome, cohesin complex, frameshift variant, congenital heart disease, whole-exome sequencing, X-linked disorder, structural modeling

## Abstract

**Background:** Mullegama–Klein–Martinez syndrome (MKMS; OMIM #301022) is an X-linked cohesinopathy caused by pathogenic variants in *STAG2*, which encodes a subunit of the cohesin complex responsible for chromosomal segregation and transcriptional regulation. Individuals typically present with developmental delay, microcephaly, dysmorphic features, and variable congenital anomalies, though complex cardiac malformations are uncommon. **Case Presentation:** We report a female infant presenting on the first day of life with complex congenital heart disease, including pulmonary atresia, double-outlet right ventricle, large subaortic ventricular septal defect, and patent ductus arteriosus. She exhibited intrauterine growth restriction, mild craniofacial dysmorphism, and left upper-extremity hypotonia. Stepwise genetic evaluation revealed a de novo likely pathogenic *STAG2* frameshift variant, c.2972_2975dup (p.His992Glnfs*11), identified by rapid trio whole-exome sequencing. This variant truncates the C-terminal domain critical for cohesin binding. A 3D structural model generated by SWISS-MODEL demonstrated disruption of β-strand and loop conformations within this domain, consistent with loss of cohesin complex stability. **Conclusions:** This case expands the phenotypic spectrum of *STAG2*-related MKM and highlights the role of *STAG2* in cardiac development. Recognition of such presentations supports the inclusion of *STAG2* in the differential diagnosis for complex congenital heart disease and underscores the diagnostic utility of rapid trio exome sequencing in neonatal care. The utility of 3D protein modeling to illustrate structural consequences of truncating variants provides valuable insight into variant pathogenicity and supports precision diagnosis in cohesinopathies.

## 1. Introduction

Cohesinopathies are a heterogeneous group of developmental disorders caused by pathogenic variants in genes encoding subunits or regulators of the cohesin complex, including *NIPBL*, *SMC1A*, *SMC3*, *RAD21*, and *STAG2* [1,2]. The cohesin complex plays a central role in sister-chromatid cohesion, DNA repair, and transcriptional regulation during embryogenesis [3,4]. Disruption of cohesin function leads to multisystem developmental abnormalities characterized by growth restriction, variable intellectual disability, and congenital malformations [2,3,5,6].

*STAG2* encodes the stromal antigen 2 protein, an essential component of the cohesin complex. Loss-of-function variants in *STAG2* cause Mullegama–Klein–Martinez syndrome (MKMS)**,** an X-linked disorder first described by Mullegama and colleagues in 2017 [1]. Affected individuals typically present with developmental delay, hypotonia, microcephaly, dysmorphic facial features, and variable congenital anomalies, including cardiac, skeletal, and genitourinary defects [1,2,7]. Although fewer than 40 cases have been reported to date, accumulating evidence indicates that *STAG2*-related disease may be under-recognized and exhibit wide phenotypic variability [8,9].

Congenital heart defects (CHDs) are among the most frequent malformations associated with cohesinopathies. CHDs occur in approximately 1% of live births and are commonly linked to chromatin-modifying and transcription-regulating genes [10,11]. Conotruncal anomalies, such as truncus arteriosus and pulmonary atresia, arise from perturbations in cardiac neural-crest and outflow-tract development and are frequently observed in chromatin-remodeling disorders [12,13]. Recent large-scale genomic studies have identified *STAG2* and other cohesin subunits among candidate genes for syndromic and isolated CHDs, supporting a critical role of cohesin in cardiac morphogenesis [14,15,16].

Emerging data indicate that *STAG2* variants can lead to both syndromic and isolated presentations of congenital heart disease, with truncating mutations producing dosage-sensitive phenotypes and variable expressivity [1,2,4]. The present report describes a female infant with a de novo *STAG2* frameshift variant who presented neonatally with severe conotruncal malformations—a phenotype rarely described in the literature. This case underscores an underrecognized aspect of cohesinopathy-associated organ involvement, thereby expanding the clinical and structural spectrum of Mullegama–Klein–Martinez syndrome (MKMS) and demonstrating the diagnostic value of integrating molecular analysis with 3D structural modeling to elucidate variant pathogenicity.

## 2. Case Presentation

The proband, AT, is a Hispanic female first evaluated by Genetics on day 1 of life (born in June 2025) for congenital cardiac anomalies identified on postnatal echocardiography. She was born at 37 + 2 weeks by spontaneous vaginal delivery to a 33-year-old G3P1011 mother. The pregnancy was detected early; the mother denied vaginal bleeding or major illness but reported a self-limited febrile episode at ~12 weeks’ gestation lasting 2–3 days, treated with acetaminophen. Routine prenatal screening, including NIPT for common aneuploidies, was negative. A fetal echocardiogram one month before delivery suggested a conotruncal malformation, suspicious for truncus arteriosus versus pulmonary atresia.

At birth, anthropometrics indicated intrauterine growth restriction: weight 2190 g (4 lb 13 oz), length 44 cm (17.3 in), and head circumference 30.5 cm (12.0 in)—all <1st percentile for gestational age. The immediate neonatal course was notable for cyanosis and hypoxemia. Echocardiography demonstrated complex congenital heart disease comprising pulmonary atresia, double-outlet right ventricle (DORV), a large subaortic VSD, an ostium secundum ASD, a moderate PDA supplying confluent branch pulmonary arteries, suspected MAPCAs, mild right-ventricular hypoplasia, and moderate left-ventricular hypertrophy. She was started on prostaglandin E_1_ (Prostin) shortly after birth and supported with CPAP for hemodynamic stabilization.

Head ultrasonography showed no acute intracranial abnormality aside from a tiny right choroid-plexus cyst, and abdominal ultrasound was unremarkable except for trace perihepatic fluid. Genetics was consulted on day 1 of life. On examination, the newborn had dysmorphic craniofacial features, including a narrow forehead, up-slanting palpebral fissures with epicanthal folds, large posteriorly rotated ears, and a thin upper lip. Family history was notable for a 14-year-old brother with growth-hormone deficiency and short stature and a paternal cousin who underwent surgical repair of a congenital heart defect at two years of age. The parents were healthy, non-consanguineous, and of Mexican (father) and Guatemalan–Mexican (mother) ancestry. No other relatives were known to have developmental delay, intellectual disability, or structural anomalies.

Initial genetic testing—including chromosomal microarray and a congenital heart-disease gene panel—was negative. Repeat postnatal echocardiography confirmed persistent pulmonary atresia with a large subaortic VSD, ASD, and a PDA supplying confluent pulmonary arteries, with preserved biventricular function. Given the combination of multiple cardiac malformations and subtle dysmorphic features, rapid trio whole-exome sequencing (rWES) was pursued through Prevention Genetics to clarify the genetic etiology

## 3. Genetic Testing and Findings

Rapid trio whole-exome sequencing (rWES) was performed by Prevention Genetics (Marshfield, WI, USA, Accession number RQ7357873) on the Illumina NovaSeq 6000 platform (Illumina Inc., San Diego, CA, USA), achieving >98% coverage at ≥20× depth. Variant calling and annotation were conducted through the Prevention Genetics clinical pipeline using GRCh38 reference. The analysis identified a heterozygous de novo frameshift variant in STAG2 (NM_001042749.2): c.2972_2975dup (p.His992Glnfs*11). This variant was absent from ClinVar and gnomAD databases and is predicted to cause a premature stop codon leading to loss of function of the cohesin subunit STAG2. According to ACMG/AMP criteria, the variant was classified as likely pathogenic. Parental testing confirmed its de novo origin. The molecular findings, in conjunction with the patient’s clinical presentation, established the diagnosis of X-linked Mullegama–Klein–Martinez syndrome (MKMS).

## 4. Management and Outcome

Following discharge, the infant underwent a modified Blalock–Taussig shunt and patent ductus arteriosus ligation at age of two months old. The postoperative course was uncomplicated, and ventricular function remained preserved. During the first months of life, her growth parameters continued below the first percentile for both weight and length. She developed mild left-arm hypotonia while hospitalized, which showed gradual improvement after discharge.

By four months of age, she demonstrated appropriate early social and visual responses, including a social smile, sustained eye contact, and visual tracking of objects, although she had not yet achieved full head control. Feeding intolerance persisted, characterized by frequent emesis requiring nasogastric-tube supplementation, and a possible milk-protein intolerance was suspected.

At seventeen weeks of age, she remained clinically stable and continued to receive multidisciplinary follow-up with cardiology, neurology, otolaryngology, gastroenterology, and the Inland Regional Center early-intervention program. Dysmorphic craniofacial features were unchanged, consisting of a narrow forehead, up-slanting palpebral fissures with epicanthal folds, large posteriorly rotated ears, and a thin upper lip. Neurological examination showed mild residual left-arm hypotonia but overall good alertness and activity. Echocardiography demonstrated satisfactory postsurgical anatomy with preserved biventricular function. A brain MRI obtained at seven weeks of age was normal.

## 5. Discussion

### 5.1. Severe Cardiac Phenotype in MKMS

Since the initial description by Mullegama et al., fewer than 40 individuals with pathogenic *STAG2* variants have been reported, with nearly all cases involving affected females and only rare male cases, likely due to male lethality or severe phenotypes [1,7,8,17]. Affected females typically present with variable combinations of developmental delay, microcephaly, intrauterine growth restriction, hypotonia, dysmorphic facial features, and multisystem involvement, including congenital anomalies of the heart, kidneys, and genitourinary tract [1,7,8]. However, the majority of published cases describe either mild or isolated cardiac anomalies, such as atrial or ventricular septal defects, with complex congenital heart disease being uncommon in this population [1,4,7,8].

In contrast, the current patient presented on the first day of life with severe conotruncal heart defects—pulmonary atresia, double-outlet right ventricle, large subaortic ventricular septal defect, and patent ductus arteriosus—requiring early surgical palliation. Such early and complex cardiovascular involvement is rarely reported among females with STAG2 variants, underscoring the exceptional severity of this case within the context of Mullegama–Klein–Martinez syndrome [1,4,8]. Notably, unlike many previously reported individuals who exhibited microcephaly or structural brain anomalies, this patient’s brain MRI was normal. She also demonstrated milder neurodevelopmental delay than typically described, consistent with the variable expressivity observed in STAG2-related disease [1,8].

The combination of severe congenital heart disease, early hypotonia, and intrauterine growth restriction in this patient expands the phenotypic spectrum of Mullegama–Klein–Martinez syndrome and supports a broader appreciation of cohesinopathy-related organ involvement.

Phenotypic diversity among reported cases may reflect differences in X-inactivation patterns, tissue-specific mosaicism, or modifying genetic factors [8]. Because *STAG2* is located on the X chromosome, random or skewed X-chromosome inactivation (XCI) can substantially influence disease expressivity in females with *STAG2*-related cohesinopathies. Skewing that favors inactivation of the mutant allele may mitigate neurological involvement, whereas preferential inactivation of the wild-type allele may lead to more pronounced features. This mechanism likely contributes to the wide spectrum of neurodevelopmental severity described in affected females and may explain the relatively mild neurological findings in the current case [1,8,17].

This case highlights the need for individualized clinical surveillance and further delineation of genotype-phenotype correlations in STAG2-associated disorders [1,4,8]. This case emphasizes that even de novo *STAG2* truncating variants can produce markedly different organ-specific outcomes, from mild developmental delay to life-threatening cardiac malformations, highlighting the need for individualized clinical surveillance and long-term follow-up.

In summary, most published STAG2 MKMS cases present with global developmental delay, microcephaly, hypotonia, and mild craniofacial dysmorphism. Cardiac defects are infrequent and typically limited to atrial or ventricular septal defects. The current case is distinguished by a severe conotruncal defect (pulmonary atresia, double-outlet right ventricle, large VSD, and PDA), which has not been previously reported in STAG2 MKMS, thereby expanding the recognized cardiac phenotype (see Table 1). This underscores the importance of comprehensive molecular and phenotypic characterization in rare cohesinopathies. This comparison highlights the unique cardiac findings in the present case and situates them within the broader spectrum of STAG2-related MKMS.

### 5.2. Genetic Insights

*STAG2* is located on chromosome Xq25 and encodes a 1229-amino-acid protein that forms a core component of the cohesin complex together with SMC1A, SMC3, and RAD21. This complex is crucial for sister-chromatid cohesion, DNA repair, and transcriptional regulation during development [4]. The C-terminal domain of STAG2 interacts directly with RAD21 and SA1/SA2 subunits, stabilizing the cohesin ring that topologically encircles DNA to maintain chromosomal integrity and orchestrate gene expression [1,2]. The N-terminal region contains multiple Armadillo (ARM) or HEAT repeats, which facilitate interactions with the cohesin core subunits SMC1A and SMC3. The central STAG domain forms the structural core of the protein and mediates direct binding to RAD21, thereby stabilizing the cohesin ring and ensuring proper chromosomal segregation [4].

Pathogenic *STAG2* variants, most often loss-of-function mutations—including nonsense, frameshift, or splice-site changes—constitute the main molecular mechanism underlying Mullegama–Klein–Martinez syndrome (MKMS)**.** These alterations typically result in haploinsufficiency, destabilizing the cohesin complex and impairing DNA binding and chromatin architecture [7,8]. Functional and structural modeling studies demonstrate that such variants disrupt key protein–protein and protein–DNA interactions, leading to altered transcriptional networks and developmental dysregulation [4,18].

**Figure 1:**
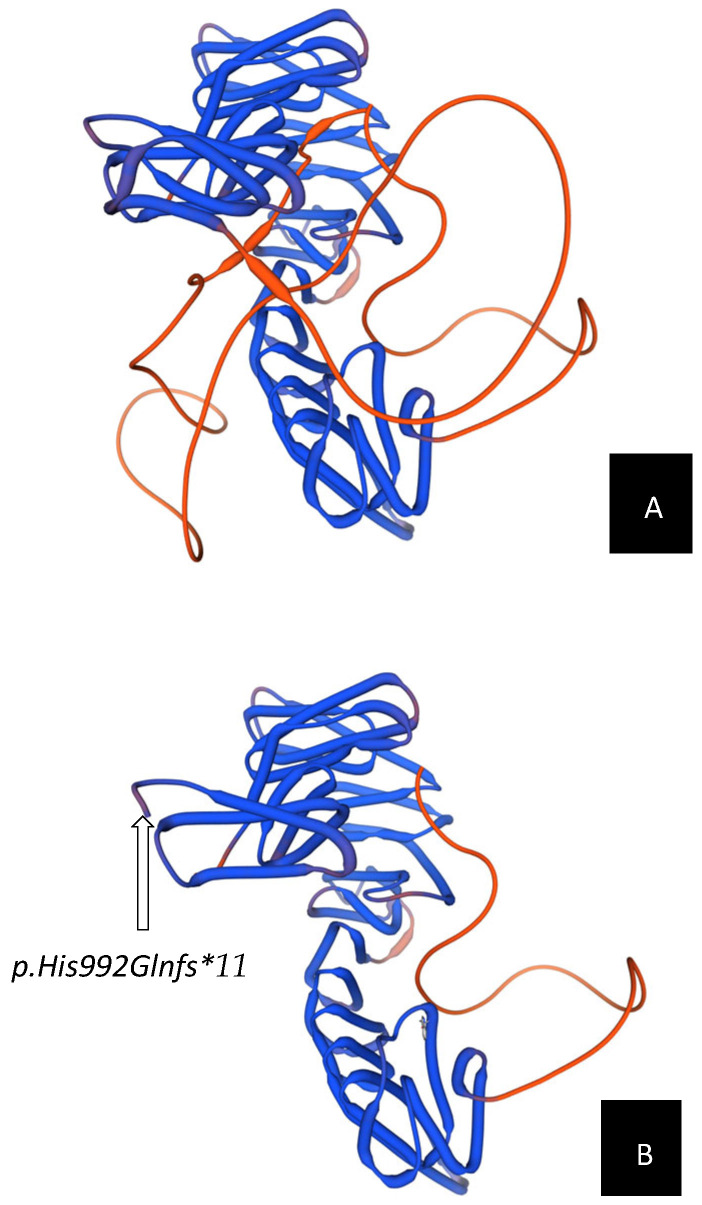
Structural comparison between wild-type and mutant *STAG2* protein models. Three-dimensional modeling of the *STAG2* protein was performed using the SWISS-MODEL server (https://swissmodel.expasy.org/). (**A**) Predicted 3D structure of the wild-type *STAG2* protein, shown in blue. The intact β-strand and loop arrangement within the C-terminal domain demonstrates a stable tertiary fold with a characteristic horseshoe-like configuration, which is critical for cohesin complex assembly and chromosomal segregation. The flexible loop region is highlighted in orange, reflecting normal conformational dynamics. (**B**) Predicted 3D structure of the mutant *STAG2* protein carrying the c.2972_2975dup (p.His992Glnfs*11) variant. The frameshift results in premature truncation of approximately 20% of the C-terminal region, causing local structural distortion and loss of the STAG domain, including the loop region that normally mediates interaction with RAD21. The model illustrates disruption of β-strand and loop elements within the C-terminal SA_C domain, which is predicted to destabilize the *STAG2–RAD21* cohesin interface.

In the current case, the de novo frameshift variant c.2972_2975dup (p.His992Glnfs*11) truncates the final 237 amino acids of STAG2. Protein structure prediction was performed using the SWISS-MODEL online server to evaluate the impact of the *STAG2* truncating variant on protein conformation. The homology model was generated using the human RAD21–STAG2 crystal structure (Protein Data Bank ID: 4PJW) as a template, which shares approximately 80% sequence identity with the modeled STAG2 C-terminal domain. This template defines the RAD21-interacting SA_C region and has been used in prior computational analyses of *STAG2* variants [4]. The resulting model illustrates the predicted disruption of β-strand-loop configuration within the cohesin-binding domain, confirmed the loss of tertiary stability and disruption of intramolecular contacts essential for cohesin loading. This structural alteration likely prevents proper cohesin-ring assembly, resulting in downstream transcriptional dysregulation during embryogenesis (See Figure 1). The variant’s de novo origin and absence from population databases such as gnomAD satisfy ACMG/AMP criteria (PVS1 + PS2 + PM2), supporting a likely pathogenic classification.

Specifically, for the cardiac anomalies, disruption of STAG2 likely perturbs cohesin-mediated chromatin looping and transcriptional control of cardiac developmental genes. The cohesin complex, through its interaction with CTCF and NIPBL, regulates the spatial organization of chromatin domains critical for cardiac neural crest cell migration and outflow tract formation [4,19,20,21]. When STAG2 function is lost, chromatin loops are destabilized, leading to dysregulation of transcriptional networks such as TBX1, NKX2-5, and HAND2—genes required for proper conotruncal development. This results in defective migration and differentiation of cardiac neural crest cells and secondary heart field progenitors, culminating in conotruncal cardiac anomalies [19,21,22,23].

Consistent with previous reports [1,2,18], this loss-of-function mechanism explains the patient’s phenotype—characterized by congenital heart disease, growth restriction, and mild dysmorphism—typical of MKMS in heterozygous females. Truncating *STAG2* variants appear fully penetrant, although clinical expressivity may vary with X-inactivation skewing and other epigenetic modifiers [8]. Collectively, these findings underscore the critical role of STAG2 in cohesin function and highlight how integrative molecular and 3D structural analyses can clarify variant pathogenicity in cohesinopathies.

Although our 3D modeling using SWISS-MODEL helps visualize the structural impact of the C-terminal truncation, these findings remain predictive. Functional confirmation through in vitro assays or cellular models is required to validate the predicted loss of cohesin-binding stability.

### 5.3. Therapeutic Considerations

Currently, no disease-specific therapy exists for Mullegama–Klein–Martinez syndrome (MKMS), and management remains primarily supportive and multidisciplinary. Treatment should be tailored to each patient’s individual clinical manifestations, with care coordination across multiple specialties. Multidisciplinary surveillance is essential, incorporating cardiology, neurology, endocrinology, audiology, and clinical genetics, given the potential for evolving multisystem involvement [2,18,24].

In the present case, the patient required early surgical palliation with a modified Blalock–Taussig shunt and patent ductus arteriosus ligation to ensure survival. Postoperative management focused on nutritional optimization, control of hypotonia, and developmental progress monitoring. Early initiation of physical, occupational, and feeding therapies has been crucial for improving neurodevelopmental outcomes and addressing feeding intolerance.

Although experimental therapeutic approaches such as cohesin-modulating agents or PARP inhibitors have been proposed, they remain preclinical and are not currently applicable to patient care [20,21,22,23,24]. Therefore, the management of *STAG2*-related cohesinopathies should emphasize evidence-based, multidisciplinary follow-up, including regular cardiology assessments, developmental evaluations, and early interventional services. Comprehensive longitudinal monitoring is vital for optimizing outcomes, detecting emerging comorbidities, and coordinating supportive care.

Genetic counseling should be offered to families regarding the X-linked inheritance pattern, recurrence risk, and reproductive options. A precise molecular diagnosis not only informs prognosis and medical management but also facilitates proactive surveillance for potential complications and supports family planning decisions.

## 6. Conclusions

This report describes a female infant presenting from birth with complex conotruncal heart disease, intrauterine growth restriction, and subtle craniofacial features, in whom rapid whole-exome sequencing identified a de novo frameshift variant in STAG2 (c.2972_2975dup; p.His992Glnfs*11). The variant truncates the C-terminal cohesin-binding region of STAG2, and three-dimensional structural modeling using SWISS-MODEL demonstrated marked distortion of the β-strand and loop configuration within this domain, supporting a loss-of-function mechanism consistent with Mullegama–Klein–Martinez syndrome.

This case broadens the phenotypic spectrum of STAG2-related cohesinopathy by illustrating that severe congenital heart malformations can represent the predominant neonatal manifestation, even before neurodevelopmental abnormalities emerge. It also highlights the diagnostic utility of rapid genomic testing in critically ill infants with complex congenital anomalies. The integration of computational modeling and in silico structural prediction—as demonstrated in this case through SWISS-MODEL—provides functional evidence supporting variant pathogenicity. As genomic technologies become increasingly embedded in neonatal intensive care and pediatric cardiology practice, combining molecular diagnosis with protein-structure modeling represents a powerful approach to elucidate disease mechanisms in rare variants. Continued reporting of molecularly and structurally characterized STAG2 cases will further refine genotype–phenotype correlations and enhance clinical recognition and management of this emerging X-linked cohesinopathy.

## Figures and Tables

**Table 1:** STAG2 related disorder phenotype comparison.

Case/Reference	STAG2 Variant	Developmental Delay	Microcephaly	Hypotonia	Craniofacial Dysmorphism	Cardiac Anomalies	Other Congenital Anomalies	Reference
Mullegama et al., 2017 (Index)	c.205C>T (p.Arg69*)	Global delay	Yes	Yes	Microtia, facial dysmorphism	ASD, VSD	Hearing loss, ADHD	[1]
Schmidt et al., 2022	Splice variant	Global delay	Yes	Hemihypotrophy	Supernumerary nipples, facial dysmorphism	None reported	Epilepsy	[8]
Lehalle et al., 2017 (Cohort)	Deletion/frameshift/missense	Mild-moderate delay	Mild (4/17)	Variable	Wide mouth, deep-set eyes	None reported	Epilepsy (7/17)	[7]
Yuan et al., 2019 (CES cohort)	De novo SNVs/indels	Mild-moderate delay	Variable	Variable	Overlapping features, not classic CdLS	Septal defects (rare)	Variable	[2]
Current Case	c.2972_2975dup (p.His992Glnfs*11)	Global delay	Yes	Left upper-extremity	Mild craniofacial dysmorphism	Pulmonary atresia, DORV, large VSD, PDA	IUGR	

## Data Availability

The original contributions presented in the study are included in the article further inquiries can be directed to the corresponding author.
